# Clinical Characteristics, Quality of Life, and Risk Factors of Amputation Stump Skin Disease and Stump Fungal Infection in Adult Amputees in Shanghai, China

**DOI:** 10.3389/fmicb.2022.868431

**Published:** 2022-04-25

**Authors:** Yanqiao Li, Longwen He, Xiangting Lu, Qian Du, Shijun Yu, Xin Huang

**Affiliations:** ^1^Department of Dermatology, School of Medicine, Tongji Hospital, Tongji University, Shanghai, China; ^2^Shanghai Hebin Rehabilitation Hospital, Shanghai, China; ^3^Shanghai Rehabilitation and Vocational Training Center for the Disabled, Shanghai, China; ^4^Department of Dermatology, Henan Provincial People’s Hospital, People’s Hospital of Zhengzhou University, People’s Hospital of Henan University, Zhengzhou, China; ^5^Department of Dermatology, School of Medicine, Shanghai Skin Disease Hospital, Tongji University, Shanghai, China; ^6^Otto Bock (China) Industries Co., Ltd., Shanghai, China

**Keywords:** quality of life, risk factors, amputation stump skin disease, fungal infection, amputees

## Abstract

**Introduction:**

The stump site of amputees is clinically vulnerable and prone to various skin diseases. Data regarding the impact on quality of life (QoL) of amputees with amputation stump skin disease (ASSD) and risk factors of ASSD and stump fungal infection in the Shanghai area are yet unknown.

**Objective:**

This study aims to evaluate the QoL of amputees with ASSD and explore the risk factors of ASSD and stump fungal infection in the Shanghai area.

**Methodology:**

A total of 104 amputees from Shanghai Hebin Rehabilitation Hospital, Otto Bock (China) Industries Co., Ltd., Shanghai Tongji Hospital, and Shanghai Rehabilitation and Vocational Training Center for the Disabled were enrolled in this study. We collected demographic, clinical, and skin fungal examination data from these amputees from April 2015 to May 2021. Dermatology life quality index (DLQI) questionnaire was used to evaluate the QoL. The risk factors for ASSD and fungal skin infection were analyzed by the univariate analyses.

**Results:**

The median age of the 104 amputees was 57.9 ± 11.9 years with an average amputation time of 17.7 ± 15.1 years, and 73% of cases were men. The mean DLQI score of amputees with ASSD was13.6, suggesting the severe impairment of QoL. Among amputees, 41 (39.4%) had confirmed ASSD, of whom 24 (58.5%) suffered from fungal skin infection and the remaining were subjected to intertriginous dermatitis and eczema (22%), cutaneous keratosis (12.2%), and others (7.3%). *Aspergillus* (50.0%) was the most common species. The other fungal organisms included *Trichophyton rubrum* (33.3%), *Candida krusei* (8.3%), *T. mentagrophytes* (4.2%), and *C. albicans* (4.2%). ASSD rather than non-ASSD was more common in men (80.4%) and summer (46.3%). Summer (OR = 3.31, 95% CI = 1.19–9.17) was an established risk factor for ASSD compared to spring. The daily artificial limb wearing time > 8 h was associated with stump fungal infection.

**Conclusion:**

The QoL of amputees with ASSD was severely affected and the ASSD was characterized by fungal infection (tinea), intertriginous dermatitis, eczema, and skin keratosis. Summer and daily prosthesis wearing > 8 h was a risk factor for ASSD. *Aspergillus* was the most common fungal species, especially when the stump was exposed in summer.

## Introduction

The World Health Organization views disability as a universal human health problem (Neal-Boylan et al., 2021). More than 1 billion people, i.e., around 15% of the global population, have some or the other form of disability (The Lancet Public Health, 2021). In the United States, there are an estimated 61 million people with disability (PWD) (French-Lawyer et al., 2021). The most common physical disability is a result of limb amputation that is typically performed as a life-saving procedure in the management of victims of natural disasters, all types of accidents, illnesses such as peripheral vascular diseases, diabetes mellitus, and malignancies (Moxey et al., 2011; Penn-Barwell, 2011; Yaşar et al., 2017). However, amputation changes the body structure and influences body activities and participation (Pezzin et al., 2000; Burger and Marincek, 2007) and leads to social and psychological dysfunction (Cervelli et al., 2008). The use of a prosthesis is one of the options for rehabilitation after amputation. Prostheses can be very helpful for mobility and participation and can improve the psychosocial function of amputees. Nonetheless, disability is often a neglected issue in public health, and people who have undergone amputation continue to experience profound inequalities in health and wellbeing outcomes. Furthermore, the restrictions resulting from amputation and the inability to adapt to the use of the artificial limb in everyday life can also negatively impact the QoL of amputees (Sinha et al., 2014). In addition, comorbidities, older age, unemployed status, and residual stump pain are reportedly significant predictors that negatively influence amputees’ QoL (Sinha et al., 2014).

The stump site of an amputee is a fragile cutaneous environment that is inclined to ASSD because of many variables such as the skin condition alteration, neurovascular structure damage, and lymphatic drainage barrier (Buikema and Meyerle, 2014). ASSD is a disease with high incidence in amputees especially in some of the amputees who wear prostheses. A prior study noted that the prevalence of ASSD was up to 73.9% in amputees (Koc et al., 2008), and the most common clinical symptoms were burning sensation, erythema, and desquamation (Dudek et al., 2005). Buikema and Meyerle (2014) demonstrated that lower-limb amputees in the United States exhibited skin problems, wherein the most common skin disease was contact dermatitis (Buikema and Meyerle, 2014). A clinical observation study revealed that the common skin diseases in patients with amputation were eczema dermatitis, contact dermatitis, infections, cysts, and ulcers (Cheng and Song, 2001). Fungal infection at the stump sites was also found due to excessive perspiration and lack of moisture evaporation. The presence of ASSD may lead to prosthesis abandonment, which has a negative influence on the physical and mental health and the QoL of amputees (Buikema and Meyerle, 2014).

In China, there is little research on ASSD. According to the latest statistical bulletin of the Shanghai Disabled Persons’ Federation, there were 595,000 disabled people in Shanghai by the end of 2020, which increased by 17,000 over the last year (Shanghai Disabled Persons’ Federation, 2021). Disability and ASSD are significant obstacles to the disabled community. Little is known about the epidemiology, QoL, and predictors for occurrence of ASSD in patients with limb amputation in Shanghai, China. Therefore, we aimed to investigate the relationship between amputation and QoL as well as ASSD and analyze the role of sociodemographic, medical, and amputation-related risk factors.

## Materials and Methods

### Study Design and Participants

This multi-center, cross-sectional study included 104 amputees from Shanghai Hebin Rehabilitation Hospital, Otto Bock (China) Industries Co., Ltd., Shanghai Tongji Hospital, and the Shanghai Rehabilitation Vocational Training Center for the Disabled. The enrollment time was from April 2015 to May 2021. ASSD was diagnosed by two experienced dermatologists. The study was approved by the institutional ethics committee (K-W-2022-002).

### Data Collection

We collected data including demographic information (age, sex, education level, amputation reason, amputation time, viability, and prosthesis-wearing time), clinical characteristics (stump photographs, amputation sites, disability level, and ASSD onset season), and laboratory outcomes (fungal microscopy and culture from skin scale). The disability level is divided into four classes according to China’s disability classification and grading standard (GB/T26341-2010) (Hohhot Disabled Persons’ Federation, 2021). The main criteria for the classification of physical disability are as follows. Level I: amputees do not possess the ability to carry out activities of daily living independently. Level II: amputees are basically unable to achieve daily activities independently. Level III: amputees can partially realize daily activities alone. Level IV: amputees are basically able to complete daily activities alone. Stump skin fungal examination data collection was performed in the group of patients with confirmed ASSD. The collection of skin scales at the rash sites was conducted as follows. After local disinfection, the inspector scraped off the skin scraps from the rash with a blunt blade, then placed the specimen on the glass slide, added a drop of 10% KOH solution, covered the cover glass, and heated it slightly on the flame of the alcohol lamp. After the specimen was dissolved, the microscopic examination was made to check whether hyphae and spores were observed. The fungal microscopy result was considered positive when the fungal mycelium and significant spores were seen under the microscope. Meanwhile, all the specimens were inoculated in Sabouraud’s dextrose agar medium and incubated at 28°C for two weeks to identify the fungal species. The species were verified based on morphological characteristics and biochemical test methods by using standard taxonomic keys (Qiao et al., 2019).

### Investigation of Impact on Quality of Life of Amputees *via* Dermatology Life Quality Index Scale

One of the aims of this study was to assess the impact of amputation stump on ASSD amputees’ QoL by using the Dermatology Life Quality Index (DLQI) questionnaire (Finlay and Khan, 1994). The data were collected by using a face-to-face questionnaire (Table 1). The DLQI is a widely used dermatology-specific tool to assess health-related QoL, which includes ten-question items and involves six dimensions with four possible responses to each item (0: not at all, 1: a little, 2: a lot, and 3: very much). Responses are calculated for the total DLQI score (0–30). Higher DLQI scores mean poorer QoL. A score of 0–1 indicates avoid effect of QoL, a score of 2–5 means a mild impact of QoL, a score of 6–10 means a moderate impact of QoL, a score of 11–20 means a severe impact of QoL, and a score of 21–30 means a very severe impact of QoL. The correlation between DLQI score and clinical characteristics of amputees was also investigated.

**TABLE 1:** The dermatology life quality index questionnaire.

Question1. Over the last week, does your skin itch, tenderness, pain, tingling? (Symptoms and feelings)
Question 2. Over the last week, do you feel embarrassed or inferior due to your skin condition? (Symptoms and feelings)
Question 3. Over the last week, how does it affect your shopping, housework, and yard arrangement because of skin problems? (Daily activity)
Question 4. Over the last week, how does skin problems affect you to wear clothes? (Daily activity)
Question 5. Over the last week, how much do skin problems affect your social or leisure life? (Leisure)
Question 6. Over the last week, how much do skin problems hinder your exercise? (Leisure)
Question 7. Over the last week, have skin problems prevented you from going to work or studying (Yes or no)? If your answer is no, how has your skin problem affected your work or study in the past week? (work or study)
Question 8. Over the last week, have skin problems prevented you from interacting with loved ones, close friends, or relatives? (Personal relationship)
Question 9. Over the last week, how much do skin problems affect your sex life? (Personal relationship) Question 10. Over the last week, how much trouble did it cause you to treat your skin problems, such as making a mess or taking up a lot of your time? (Treatment)

*There are four answers to each question: “Very much” (score 3), “A lot” (score 2), “A little” (score 1), or “Not at all” (score 0). The question 7 has the choices “Yes” (score 3) or “No” (score 0). The total score ranges from 0 to 30. A score of ≤ 10 means moderate or less effect, and a score of > 10 corresponds to a severe or very severe impact on the QoL. The higher the score, the more the QoL is impaired.*

### Statistical Analysis

Continuous variables were presented as the mean ± *SD*, and categorical variables were presented as *n* (%). Fisher’s exact test or the Cochran-Mantel-Haenszel test was used to compare categorical data. Logistic regression analysis was performed for the prediction of risk factors for ASSD and fungal infection to calculate the ORs with 95% CIs. Statistical analyses were done using the SAS software V.9.4 (SAS Institute), and *P* < 0.05 was considered to indicate statistically significant differences.

## Results

### Demographics and Clinical Characteristics

The demographic and clinical characteristics are shown in Table 2. A total of 104 individuals with amputation stump [41 (39.4%) ASSD and 63 (60.6%) non-ASSD] were included in this study, of which 76 (73%) amputees were male. In the ASSD group, the proportion of male amputees was as high as 80.4%, compared with 68.2% in the non-ASSD group. The mean age of all amputees was 57.9 ± 11.9 years, and the median amputation time was 17.7 ± 15.1years. Among these amputees, the most common amputation causes were traffic accidents [36 (35.2%)], occupational injury [27 (26.4%)], diabetes mellitus [11 (10.7%)], malignant tumor [14 (13.7%)], and others [11 (10.7%)], respectively. Amputees were divided into four levels of disability, of which 47 (45.6%) amputees had grade III disability, 32 (31%) had grade IV disability, 20 (19.4%) had grade II disability, and 4 (3.8%) had grade I disability. Most amputees (89.4%) used modern prosthetics. The median daily wearing prosthesis time of the ASSD group was shorter than that of the non-ASSD group (8.7 ± 3.5 h vs. 10.3 ± 2.6 h, *P* < 0.05). In terms of self-care ability, 68% of amputees could basically take care of themselves.

**TABLE 2:** Demographic and clinical characteristics of amputees and those with or without ASSD.

Factors	All amputees *n* = 104 *n* (%)	Non-ASSD *n* = 63 *n* (%)	ASSD *n* = 41 *n* (%)
**Sex**			
Male	76 (73.08)	43 (68.25)	33 (80.49)
Female	28 (26.92)	20 (31.75)	8 (19.51)
**Age (years)**			
Mean ± *SD*	57.90 ± 11.92	57.19 ± 13.71	59.00 ± 8.50
**Amputation time (years)**			
Mean ± *SD*	17.73 ± 15.11	18.19 ± 14.91	17.00 ± 15.57
**Amputation reason**			
N (missing)	102 (2)	62 (1)	40 (1)
Traffic accidents	36 (35.29)	25 (40.32)	11 (27.50)
Occupational injury	27 (26.47)	18 (29.03)	9 (22.50)
Diabetes mellitus	11 (10.78)	5 (8.06)	6 (15.00)
Vascular disease	3 (2.94)	0 (0.00)	3 (7.50)
Malignant tumor	14 (13.74)	11 (17.74)	3 (7.50)
Others	11 (10.78)	3 (4.85)	8 (20.0)
**Disability level**			
N (missing)	103 (1)	62 (1)	41 (0)
I	4 (3.88)	1 (1.61)	3 (7.32)
II	20 (19.42)	12 (19.35)	8 (19.51)
III	47 (45.63)	23 (37.10)	24 (58.54)
IV	32 (31.07)	26 (40.94)	6 (14.63)
**Prosthetic type**			
Traditional	6 (5.77)	4 (6.35)	2 (4.88)
Modern	93 (89.42)	55 (87.30)	38 (92.68)
None	5 (4.81)	4 (6.35)	1 (2.44)
**Prosthesis wearing (h/day)**			
Mean ± SD	9.77 ± 3.06	10.38 ± 2.60	8.79 ± 3.51
**Viability**			
N (missing)	100 (4)	60 (3)	40 (1)
Basically provided	68 (68.00)	41 (68.33)	27 (67.50)
Partly provided	30 (30.00)	17 (28.33)	13 (32.50)
not provided	2 (2.00)	2 (3.34)	0 (0.00)

### Evaluation of Quality of Life in Amputees *via* the Dermatology Life Quality Index Score and Its Relationship to Demographic Data

**FIGURE 1:**
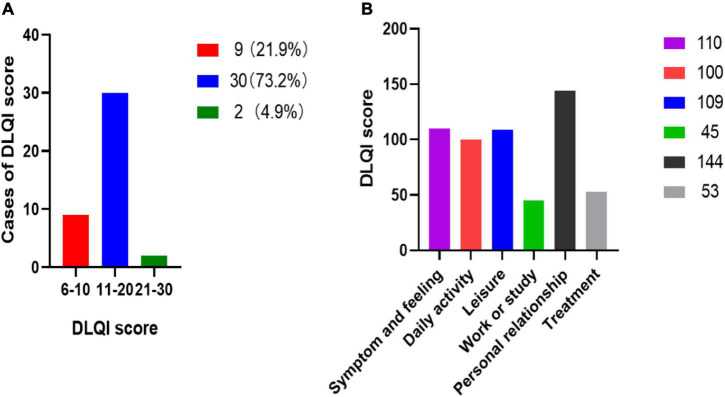
Distribution of DLQI score and six affected dimensions of QoL. **(A)** The distribution of amputees DLQI score. **(B)** DLQI score of six affected dimensions of QoL.

The QoL of amputees with ASSD was assessed by the DLQI scale. The results showed that 9 (21.9%) cases with ASSD had a moderate effect on their QoL (DLQI: 6–10), 30 (73.2%) cases with ASSD had a severe effect (DLQI: 11–20), 2 (4.9%) cases with ASSD had a very severe effect (DLQI: 21–30) (Figure 1A). The mean DLQI score was 13.6, indicating severe damage of QoL. Among the six dimensions, personal relationship (total DLQI score: 144), symptom and feeling (total DLQI score: 110), and leisure (total DLQI score: 109) were the most affected dimensions (Figure 1B). Furthermore, the correlation between the clinical characteristics data and DLQI score was investigated. There were no statistically significant differences in the influence of age, sex, amputation time, amputation site, disability level, viability, education level, and ASSD classification on QoL between DLQI ≤ 10 and DLQI > 10 (Table 3).

**TABLE 3:** Distribution of participants with ASSD *via* the DLQI score according to sociodemographic and clinical data.

Factors	Amputees with ASSD *n* (%)	Amputees with DLQI≤10 *n* (%)	Amputees with DLQI>10 *n* (%)	Statistics	*P*-value
**Sex**					
All	41 (0)	38 (0)	2 (0)	−	1.0000
Male	33 (80.49)	30 (78.95)	2 (100.00)		
Female	8 (19.51)	8 (21.05)	0 (0.00)		
**Age (years)**					
N (missing)	41 (0)	38 (0)	2 (0)	−	1.0000
≤60	24 (58.54)	23 (60.53)	1 (50.00)		
>60	17 (41.46)	15 (39.47)	1 (50.00)		
**Amputation time (years)**					
N (missing)	40 (1)	37 (1)	2 (0)	−	0.5263
≤20	28 (70.00)	26 (70.27)	1 (50.00)		
>20	12 (30.00)	11 (29.73)	1 (50.00)		
**Amputation site**					
N (missing)	41 (0)	38 (0)	2 (0)	χ^2^ = 0.13	0.9360
Left lower limb	18 (43.90)	16 (42.11)	1 (50.00)		
Right lower limb	21 (51.22)	20 (52.63)	1 (50.00)		
Upper forearm	2 (4.88)	2 (5.26)	0 (0.00)		
Bilateral lower limbs	0 (0.00)	0 (0.00)	0 (0.00)		
Others	0 (0.00)	0 (0.00)	0 (0.00)		
**Disability level**					
N (missing)	41 (0)	38 (0)	2 (0)	χ^2^ = 0.27	0.6032
I	3 (7.32)	3 (7.89)	0 (0.00)		
II	8 (19.51)	7 (18.42)	1 (50.00)		
III	24 (58.54)	23 (60.53)	1 (50.00)		
IV	6 (14.63)	5 (13.16)	0 (0.00)		
**Viability**					
N (missing)	40 (1)	37 (1)	2 (0)	χ^2^ = 0.91	0.3393
Basically provided	27 (67.50)	25 (67.57)	2 (100.00)		
Partly provided	13 (32.50)	12 (32.43)	0 (0.00)		
**Education level**					
N (missing)	40 (1)	37 (1)	2 (0)	χ^2^ = 0.01	0.9136
Primary school or below	8 (20.00)	8 (21.62)	0 (0.00)		
Junior high school	21 (52.50)	19 (51.35)	2 (100.00)		
Senior high school or technical school	11 (27.50)	10 (27.03)	0 (0.00)		
University and above	0 (0.00)	0 (0.00)	0 (0.00)		
**ASSD classification**					
N (missing)	41 (0)	38 (0)	2 (0)	χ^2^ = 7.07	0.0697
Tinea corporis	24 (58.54)	23 (60.53)	0 (0.00)		
Intertriginous dermatitis and eczema	5 (12.20)	5 (13.16)	0 (0.00)		
Cutaneous keratosis	9 (21.95)	8 (21.05)	1 (50.00)		
Others	3 (7.32)	2 (5.26)	1 (50.00)		

### Clinical Manifestations and Classification of Amputation Stump Skin Disease

**FIGURE 2:**
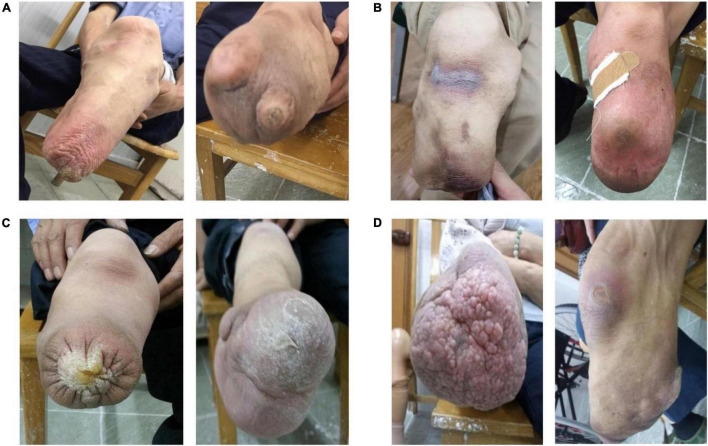
Representative clinical lesion manifestations and patient distribution of ASSD. **(A)** Stump tinea corporis with fungal infection of *Trichophyton rubrum* (left) and *Aspergillus* (right). **(B)** Intertriginous dermatitis (left) and eczema (right). **(C)** Cutaneous keratosis. **(D)** Verrucous hyperplasia (left) and skin ulceration (right).

In this study, 41 (39.4%) amputees had confirmed ASSD among the amputees with a mean age of 59.0 ± 8.5 years. The type of ASSD in these cases included tinea corporis (fungal infection) (Figure 2A), intertriginous dermatitis and eczema (Figure 2B), cutaneous keratosis (Figure 2C), and verrucous hyperplasia and ulceration (Figure 2D). Tinea corporis accounted for the majority of cases (58.5%), followed by intertriginous dermatitis and eczema (22%), cutaneous keratosis (12.2%), and others (7.3%).

### Prediction of Risk Factors for Amputation Stump Skin Disease and Stump Skin Fungal Infection Identified by Logistic Regression Analysis

The risk factors associated with ASSD and stump fungal infection are shown as Table 4. The influence in ASSD amputees with prosthesis wearing time < 8 h was more than those with prosthesis wearing time > 8 h (OR: 3.00, 95% CI: 1.05–8.54, *P* < 0.05), which implied prosthesis wearing was a potential risk factor for ASSD. Meanwhile, summer was identified as an important risk factor, and ASSD was 3.31-times more likely to occur in summer than in spring (OR: 3.31, 95% CI: 1.19–9.17, *P* < 0.05). Disability level was identified as a significant protective factor for ASSD and stump fungal infection (*P* < 0.05). Age, sex, amputation time, prosthetic type and cleaning, and DLQI scores were not significant risk factors of ASSD as well as fungal infection (all *P* > 0.05).

**TABLE 4:** Predictors of risk factors for ASSD and skin fungal infection.

Factors	Amputees		OR (95%CI)	*P*	ASSD with fungal infection		OR (95%CI)	*P*
						
	Non-ASSD *n* (%)	ASSD *n* (%)			No	Yes		
Sex				0.1730				0.4456
Male	43 (68.25)	33 (80.49)	1.92 (0.75, 4.90)		57 (71.25)	19 (79.17)	1.53 (0.51, 4.59)	
Female	20 (31.75)	8 (19.51)	1.00		23 (28.75)	5 (20.83)	1.00	
Age (years)				0.7112				0.8090
≤ 60	34 (54.84)	24 (58.54)	1.00		45 (56.96)	13 (54.17)	1.00	
>60	28 (45.16)	17 (41.46)	0.86 (0.39, 1.91)		34 (43.04)	11 (45.83)	1.12 (0.45, 2.81)	
Disability level				0.0236				0.0187
I	1 (1.61)	3 (7.32)	1.00		2 (2.53)	2 (8.33)	1.00	
II	12 (19.35)	8 (19.51)	0.22 (0.02, 2.53)		18 (22.78)	2 (8.33)	0.11 (0.01, 1.28)	
III	23 (37.10)	24 (58.54)	0.35 (0.03, 3.59)		30 (37.97)	17 (70.83)	0.57 (0.07, 4.39)	
IV	26 (41.94)	6 (14.63)	0.08 (0.01, 0.87)		29 (36.71)	3 (12.50)	0.10 (0.01, 1.02)	
Amputation time				0.2913				0.8657
≤20	37 (59.68)	28 (70.00)	1.58 (0.68, 3.67)		50 (63.29)	15 (65.22)	1.09 (0.41, 2.88)	
>20	25 (40.32)	12 (30.00)	1.00		29 (36.71)	8 (34.78)	1.00	
Prosthesis wearing (daily)				0.0395				0.0962
≤8 h	9 (20.00)	12 (42.86)	3.00 (1.05, 8.54)		13 (23.64)	8 (44.44)	2.58 (0.84, 7.91)	
>8 h	36 (80.00)	16 (57.14)	1.00		42 (76.36)	10 (55.56)	1.00	
Viability				0.9446				0.1329
Basically provided	41 (68.33)	27 (67.50)	1.00		56 (72.73)	12 (52.17)	1.00	
Partly provided	17 (28.33)	13 (32.50)	1.16 (0.49, 2.77)		19 (24.68)	11 (47.83)	2.70 (1.02, 7.12)	
Not provided	2 (3.33)	0 (0.00)			2 (2.60)	0 (0.00)	−	
Prosthetic type				0.6366				0.8677
Traditional	4 (6.35)	2 (4.88)	1.00		4 (5.00)	2 (8.33)	1.00	
Modern	55 (87.30)	38 (92.68)	1.38 (0.24, 7.93)		71 (88.75)	22 (91.67)	0.62 (0.11, 3.61)	
None	4 (6.35)	1 (2.44)	0.50 (0.03, 7.99)		5 (6.25)	0 (0.00)	−	
Prosthesis cleaning (daily)				0.3168				0.4218
Yes	48 (96.00)	28 (90.32)	0.39 (0.06, 2.47)		58 (95.08)	18 (90.00)	0.47 (0.07, 3.01)	
No	2 (4.00)	3 (9.68)	1.00		3 (4.92)	2 (10.00)	1.00	
ASSD season				0.0100				0.1307
Spring	23 (36.51)	12 (29.27)	1.00		30 (37.50)	5 (20.83)	1.00	
Summer	11 (17.46)	19 (46.34)	3.31 (1.19, 9.17)		18 (22.50)	12 (50.00)	4.00 (1.21, 13.22)	
Autumn	12 (19.05)	7 (17.07)	1.12 (0.35, 3.58)		12 (15.00)	7 (29.17)	3.50 (0.93, 13.22)	
Winter	17 (26.98)	3 (7.32)	0.34 (0.08, 1.39)		20 (25.00)	0 (0.00)	−	

### Fungal Species Identification Outcomes of Stump Fungal Infection and the Approach Methods to Prosthesis Nursing and Dermatological Rehabilitation Guidance

**FIGURE 3:**
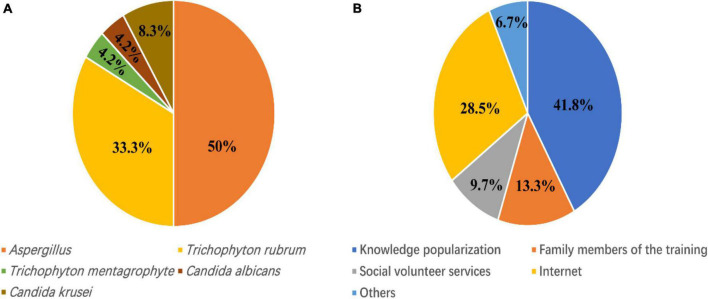
Fungal species identification outcomes and the ways to skin rehabilitation guidance**. (A)** Fungal species identification findings from 24 amputees of fungal skin infection. **(B)** The methods of prosthesis nursing and skin rehabilitation guidance.

Fungal specimen data from 24 amputees of stump fungal infection were collected. The findings are shown in Figure 3A. *Aspergillus* (50%) was the most common fungal species, followed by *Trichophyton rubrum* (33.3%), *Candida krusei* (8.3%), *T. mentagrophytes* (4.2%), and *C. albicans* (4.2%). Among ASSD, amputees received guidance on skin care and rehabilitation through the knowledge popularization (41.8%), internet (28.5%), training of family members (13.3%), social volunteer services (9.7%), and others (6.7%) (Figure 3B).

## Discussion

This study was a multicenter cross-sectional study that included 104 amputees in Shanghai with a median age of 57.9 years old. In all, 41 (39.4%) cases were diagnosed with ASSD, and the prevalence of skin problems was lower than that (77%) in a recent study in persons with a lower limb amputation (Mollee et al., 2021). The discrepancy may be attributed to differences in geographical location, sampling methods, or employed type of screening instruments. Consistent with the findings of a previous report (Buikema and Meyerle, 2014), ASSD in our study was characterized by tinea corporis, intertriginous dermatitis and eczema, verrucous hyperplasia and ulceration. Besides, in this study, skin keratosis accounted for a large proportion of ASSD, which may be related to the long disability course and chronic friction leading to skin thickening and keratosis. Furthermore, we also investigated the QoL and several possible predictive factors for ASSD and stump fungal infection among amputees in Shanghai, China. The findings could help amputees or clinicians to identify the risk factors of ASSD occurrence and stump fungal invasion early, thereby facilitating prompt preventative intervention and comprehensive stump rehabilitation.

Amputation is considered a detrimental event affecting QoL (Burçak et al., 2021). Previous studies demonstrated that lower-limb amputees had worse QoL than the general population (Asano et al., 2008; Sinha and Van Den Heuvel, 2011; Sinha et al., 2011). The DLQI was a valid and reliable scale for assessing the influence of skin problems on QoL. A higher DLQI score meant worse QoL. In this study, to our best knowledge, DLQI was used for the first time to evaluate the effect on QoL of amputees with ASSD. The mean DLQI score in our study was 13.6, suggesting a severe limitation in QoL. This DLQI score was higher than the results reported in a QoL assessment study with a mean DLQI score of 6.3 in patients with alopecia by Zhang and Zhang (2017). Meanwhile, Sendrasoa et al. (2020) reported a very poor QoL in patients with psoriasis (mean DLQI: 13.8). Another study (Davidson et al., 2010) in which the SF-36 questionnaire was used to assess QoL reported worse outcomes in upper limb amputees than in lower limb amputees. Seymour et al. (2007) also investigated the QoL using SF-36, and amputees using microprocessor-controlled knee (MPK) prostheses suffered from minimal impairment of QoL. In our study, as shown in the results, most amputees’ QoL was severely affected [30 (73.2%)]. The very severe impairment of QoL (DLQI > 20) was found in 2 (4.9%) amputees. Personal relationship, symptom and feeling, and leisure were the most affected dimensions. In addition, we also investigated the possible association between clinical characteristics and QoL. The results indicated that there was no correlation between age, sex, amputation time, amputation site, disability level, viability, education level, ASSD classification, and affected degree of QoL (DLQI ≤ 10 and DLQI > 10) in amputees with ASSD.

In our study, univariate analysis identified risk factors for ASSD and stump skin fungal infection. We found that prosthesis wearing time and summer were confirmed risk factors for ASSD. The possibility to skin disease occurrence was 3.31-times more in summer than in spring (OR: 3.31, *P* < 0.05). However, disability level was identified as a significant protective factor for ASSD and stump fungal infection. The causes of the prevalence of ASSD and stump fungal infection among amputees remain unclear, although there are several likely reasons for this. First, the trauma of amputation often results in scars, skin invaginations, and bony vegetations, making the skin more fragile and prone to skin problems. Additionally, a history of diabetes, vascular disease, and malignancy predisposes the residual limb’s skin to immune dysregulation. As an immunocompromised area, the stump skin is more prone to infection, inflammation, or even malignancies (Buikema and Meyerle, 2014). Further, the skin of and around the amputation area is not physiologically adapted to the prosthesis. The enclosed space of the prosthesis socket creates increased temperature and humidity conditions, making the amputation skin site vulnerable to opportunistic infections from bacteria, fungi, or viruses. In the present study, fungal infection (tinea) was found in 58.5% of ASSD amputees at the stump sites. It is widely accepted that tinea could cause skin erythema, scales, and itching and usually occurs at the site of the amputation or in the groin area (Buikema and Meyerle, 2014). There are four types of antifungal drugs available in clinic: azoles, echinocandins, polyenes and pyrimidines (Zhang et al., 2021). Tinea can be treated with azoles like oral fluconazole or itraconazole, as well as topical antifungal creams and powders. Moreover, a phenomenon called choke syndrome could result in verrucous hyperplasia (Mueller et al., 1995; Meulenbelt et al., 2007). Choke syndrome can result in obstruction of venous return of distal tissues and eventually the amputation skin might become indurated, which could be attenuated by adjustments to the prosthesis in pressure distribution. Hence, most amputees believed that ASSD was caused by their prostheses, and reports suggested that more than 50% of patients abandoned their prostheses for a month in the prior year (Yang et al., 2012). So, the amputees in our study in the ASSD group spent less time wearing prostheses daily on average than those in the non-ASSD group (8.79 ± 3.51 h/day vs. 10.38 ± 2.60 h/day). These findings highlighted the fact that ASSD resulted in a reduction in prosthesis use, which was consistent with the results of a previous study by Meulenbelt et al. (2011). Therefore, to achieve a good rehabilitation effect after amputation, the amputees are required to learn to identify whether the prosthesis is a good fit, and a clinical follow-up is essential for prosthesis adjustments (Buikema and Meyerle, 2014). At the same time, researchers continue to investigate ways to increase the adaptation and durability of the stump skin to reduce skin breakdown, contaminations, and inflammation on the skin weak sites (Buikema and Meyerle, 2014).

In addition to the aforementioned factors, research has suggested that folliculitis was also associated with skin diseases of the stump. The skin condition was usually worse in warmer weather because of increased perspiration, skin maceration, and friction (Buikema and Meyerle, 2014). Further, according to the research by Meulenbelt et al. (2009), washing the stump ≥ four times a week, use of walking aids and antibacterial soap, use of a liner, and smoking were provocative determinants of ASSD in lower-limb amputees. Other research studies have also shown that determinants such as the level of amputation, comorbidity, and age were found to influence the rehabilitation process of the stump and skin condition (Geertzen et al., 2001). In general, limb amputation is a life-changing event that is deemed the primary cause of global disability (Lazzarini et al., 2018). Besides mobility limitations, persons with limb amputation also experience multiple health challenges including phantom limb pain, back pain, heterotopic ossification, anxiety, and depression (Cimino et al., 2021). Therefore, with respect to disability and rehabilitation, there is growing recognition of the importance of improving the QoL of amputees (Sinha and Van Den Heuvel, 2011; Amtmann et al., 2015).

There are some limitations in our study. Although this study was performed across multicenter disability and medical centers, the sample size was limited and some information was missing. Information and selection bias may also have some effect on the results. The comparison analysis of some factors did not show significant differences, which was possibly attributed to the small sample size of cases. A larger-scale multicenter study of the amputee population will help to further determine the clinical features and risk factors of ASSD as well as skin fungi invasion. Taken together, despite the study limitations, our preliminary results contribute to understanding the impact of ASSD on amputees’ QoL. There are limited published studies on the QoL of amputees with ASSD. Thus, to our knowledge, this study for the first time explores the associated risk factors for ASSD and stump fungal infection in Shanghai, China, and contributes to providing future guidelines for the protection and maintenance of the stump skin in amputees.

## Conclusion

This present study included 104 confirmed amputees in Shanghai, China, with a median age of 57.9 and a mean DLQI score of 13.6, which suggested a severe influence on the QoL. Our work revealed that personal relationship, symptom and feeling, and leisure were the most affected dimensions of the QoL. ASSD was characterized by tinea corporis, keratosis, intertriginous dermatitis and eczema, verrucous hyperplasia, and ulceration. Prosthesis wearing time and summer were confirmed risk factors for ASSD. Meanwhile, the disability level was identified as a significant protective factor for ASSD and stump skin fungal infection. The identified fungal species were *Aspergillus*, *T. rubrum*, *C. krusei*, *T. mentagrophytes*, and *C. albicans*. The results in this study suggest that ASSD in amputees needs to be addressed, and further investigations can be focused on the prevention of ASSD and fungal skin infections in amputees in the future.

## Data Availability Statement

The original contributions presented in the study are included in the article/supplementary material, further inquiries can be directed to the corresponding author.

## Ethics Statement

This study was reviewed and approved by the Ethics Committee of Shanghai Tongji Hospital.

## Author Contributions

YL, LH, and XH contributed to conception and design of the study. SY collected the clinical samples and data. XL and QD performed clinical followed-up and data summary. YL wrote the manuscript. LH and XH contributed to revision of the manuscript. All authors have contributed to the article and approved the final manuscript.

## Conflict of Interest

SY was employed by the company Otto Bock (China) Industries Co., Ltd. The remaining authors declare that the research was conducted in the absence of any commercial or financial relationships that could be construed as a potential conflict of interest.

## Publisher’s Note

All claims expressed in this article are solely those of the authors and do not necessarily represent those of their affiliated organizations, or those of the publisher, the editors and the reviewers. Any product that may be evaluated in this article, or claim that may be made by its manufacturer, is not guaranteed or endorsed by the publisher.
